# Ust-Luga Seaport of Russia: Biological Invasions and Resting Stages Accumulation

**DOI:** 10.3390/life13010117

**Published:** 2022-12-31

**Authors:** Victor Alekseev, Natalia Sukhikh

**Affiliations:** Department of Systematics, Laboratory of Freshwater and Experimental Hydrobiology, Zoological Institute of Russian Academy of Science, Universitetskaya Emb., 1, 199034 St-Petersburg, Russia

**Keywords:** dormancy, biological invasions, resting stages, Baltic Sea, ballast water, sediments

## Abstract

This article describes the results of a three-year study of invasive species of aquatic ecosystems in the vicinity of Ust-Luga, the largest Russian seaport in the Baltic. Taking into account the great importance of the participation of marine vessels in the dispersal of invasive species, an experimental study of the seasonality of accumulation of resting stages of aquatic invertebrates in the ballast compartments of a vessel located in the Baltic Sea of the Gulf of Finland was carried out. Experiments show that the time of filling the ballast compartments in late summer and autumn poses the greatest risk for the spread of aquatic invertebrates with ship ballast water. In the Baltic Russian port, 11 invasive species of zooplankton and zoobenthos were found, which comprises 15% of the biodiversity in the samples. Copepoda demonstrated the highest presence of invasive species in class among zooplankton groups (14%) and Malacostraca among benthos groups (80%). Alien species findings correspond to the main vectors of invasive species dispersal for the Baltic Sea: North America, Indochina, and the Ponto-Caspian region.

## 1. Introduction

**Figure 1:**
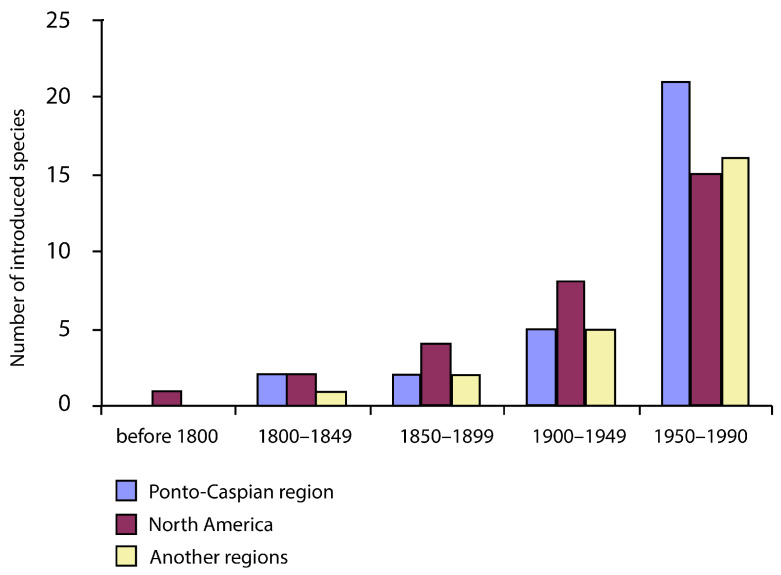
Biological invasion in Baltic Sea in 20th Century from Ponto-Caspian, North America and other regions (modified after [11]).

The dispersal of organisms across continents and oceans in aquatic ecosystems seems to have significantly accelerated in the Baltic Sea since the late 19th century, when ballast water compartments were introduced into the design of ships (Figure 1). Ballast is absolutely essential to the safe and efficient operation of ships, providing balance and stability when the cargo is empty. However, it is also becoming a cause of serious ecological, economic and health threats. Marine as well as continental aquatic species may be transported in the ballast water of ships, including pathogenic bacteria, toxic algae, invertebrates and their eggs, cysts and larvae of various species [1,2,3].

Shipping moves over 80% of the world’s commodities and transfers about 10 billion tons of ballast water each year, leading to reductions in natural barriers, such as temperature and land/oceanic masses, that previously have prevented distant dispersal of many species. As a result, whole ecosystems are changing and economic impacts can be massive [3,4,5].

A recent review on the role of ballast water and sediment in the transport of invasive species analyzed about 400 publications on this topic from 1985 to 2013 [6]. The impetus for the stream of many studies of ballast water as a vector of dispersal was the comprehensive work of James T. Carlton “Transoceanic and interoceanic dispersal of coastal marine organisms: the biology of ballast water” [7]. At the same time, most ballast research focuses on metazoans and microorganisms inhabiting ballast water. Far less attention is given to ballast sediments and resting stages.

Most planktonic stages of marine and continental species are sensitive to oxygen and pollution conditions of ship ballast water compartments. To survive in these harsh environmental conditions, dispersal stages have to be protected [8]. The resting stages of invertebrates have effective protective mechanisms that allow them to move with ballast water while maintaining viability [9]. In addition, diapause helps newcomers adapt to the specific conditions of a new environment [10].

The semi-enclosed Baltic Sea is the world’s largest brackish water sea area, which is isolated from the North Sea and north-eastern Atlantic Ocean by both geographical (sill depth 18 m, total opening less than 20 km) and ecological barriers (e.g., coldness and low salinity) [12]. However, the intensification of ship traffic has led to the overcoming of geographical barriers for other brackish species of the world. Now, the Baltic and its basin are connected with the Atlantic through the Danish straits, as well as with the Ponto-Caspian brackish seas by rivers and canals, opened in the period from 1775 to 1952 [13].

To prevent the transfer of “Harmful Aquatic Organisms and Pathogens (HAOP)” with ballast water, the International Maritime Organization developed a set of regulations and adopted the International Convention for the Control and Management of Ships’ Ballast Water and Sediments—Ballast Water Management Convention, which is scheduled to enter into force in 2024 [14]. However, these regulations do not address the issue of seasonality.

The aim of this paper was to study seasonal differences in dormant-stage production and accumulation in ship compartments and aquatic sediments in the vicinity of Ust-Luga, the largest Russian seaport in the Baltic (St Petersburg district).

Another aim of our study was to assess the proportion and origin of invasive species in plankton and benthic communities of this seaport area.

## 2. Material and Methods

The study was carried out in the area close to the seaport Ust-Luga (the Luga River and its estuary), as well as on Kotlin Island (the Gulf of Finland) and in the area of the Saint Petersburg Dam (marine community). Zooplankton, zoobenthos and sediment samples were collected using traditional tools: a Jedi net for zooplankton (25 cm open hole; mesh size 100 µm), a Petersen grab for benthos and a Sorokin stratometer for sediments. For small-sized animals (rotifers, nauplia, etc.), we used bathometer sampling and filtration (10 L via 50 µm net). All samples were collected in 3 replicates, each replicate treated separately.

**Figure 2:**
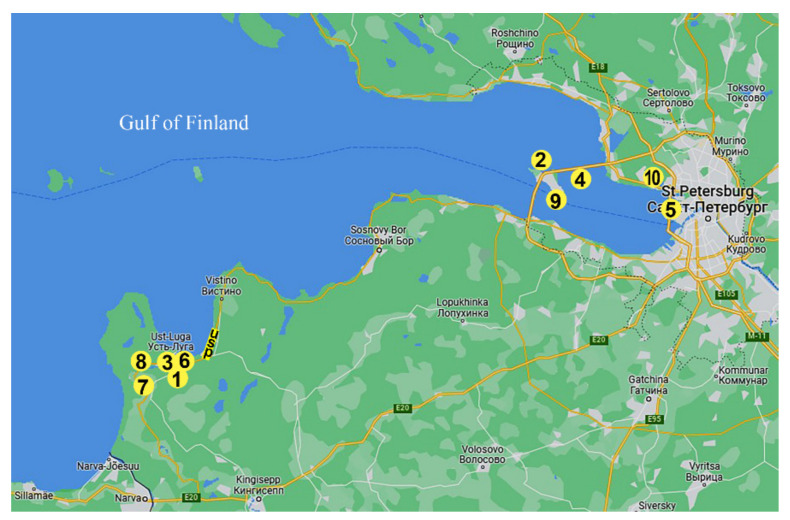
Location of sampling sites in eastern part of Gulf of Finland. USP—Ust-Luga seaport. Number of sites corresponds to Table 2 (modified from Google maps).

Samples were collected every 10th day from May to October (ice-free period) from 8 sites in 4 categories that were used for reactivation experiments (Figure 2) (Table 1).

**Table 2:** Resting-stage density and biodiversity in sediments of marine and continental waterbodies of I-IV categories (mean and range).

NN Site	Locality, (Depth, m)	Category	Density, i/m^2^	Groups Found	Dominant Species
1	Ust-Luga river port, (6–12 m)	I	23,000 (1500–40,500)	Branchiopoda Copepoda Rotifera	*Eurytemora affinis*
2	Kronshtadt port (10–18 m)	I	69,000 (1250–125,500)	Branchiopoda Copepoda Rotifera	*Eurytemora affinis* *Cercopagis pengoi*
3	Luga River estuary (4 m)	I	32,750 (1050–65,000)	Branchiopoda Copepoda	*Eurytemora affinis*
4	Neva River estuary (7 m)	I	43,500 (1250–75,000)	Branchiopoda Copepoda	*Eurytemora affinis*
5	Neva estuary near shore (1 m)	II	0		
6	Luga estuary near shore (0.5 m)	II	0		
7	Luga River (4 m)	III	6500 (150–11,200)	Branchiopoda Copepoda Insecta	*Mesocyclops leuckarti, Daphnia cucullate*
8	Temporary waterbody Ust-Luga port vicinity	IV	7914 (2340–11,700)	Branchiopoda Copepoda	*Eurytemora affinis*
9	Temporary waterbody, Cotlin Isl, Kronshtadt Port vicinity	IV	2908 (1170–4680)	Branchiopoda Copepoda Rotifera Nematoda	*Metacyclops minutus*
10	Temporary waterbody, Neva estuary	IV	18,970 (1170–37,440)	Rotifera Nematoda	Bdelloidea

### 2.1. Reactivation Protocol

Resting stages in samples collected in spring time were suspected to be almost terminated and ready for immediate reactivation. To separate dormant stages from active organisms possibly present in sediments, all samples were maintained at anoxic conditions with water saturated with CO_2_ for 1–2 months at a temperature of 4–6 °C. For reactivation, all samples were divided into equal parts. One part was placed in Petri dishes with a diameter of 10 cm, the other was placed in a beaker with a volume of 100 mL with constant aeration. Reactivated organisms were collected and counted every day for at least 2 weeks until reactivation was completed. Reactivation temperature at night 16 °C, daytime 25 °C, photoperiod L/D = 16/8. Programmable climatic chambers of the Laboratory of Experimental Entomology of the Zoological Institute in St. Petersburg were used for reactivation.

For species identification of copepods, specimens were grown to adult stages (usually 3–5 days). Nematodes, ostracods, rotifers and insect larvae were identified immediately to the nearest taxon.

All field samples were divided into 4 categories depending on the environment (marine or land) and the stability of the water level.

### 2.2. Design of Full-Scale Experiment in Ship’s Ballast Compartment

**Figure 3:**
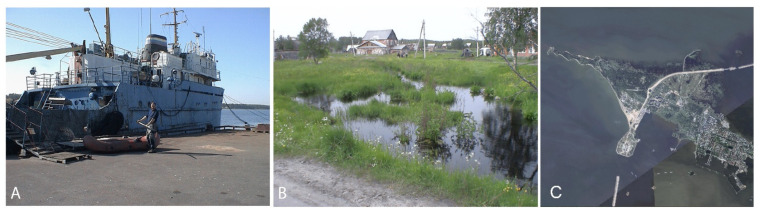
Sampling sites. (**A**)—fish vessel “Ostrov”, the Luga River estuary; (**B**)—temporary waterbody, Ust-Luga port vicinity; (**C**)—Kotlin Island, Saint Petersburg Dam area.

The ship’s ballast tank (12 tons, size about 4 × 6 m, depth 0.5 m) was rented for an experiment in real conditions for one year from the fishing vessel “Ostrov”, which was moored at the pier and operated as a fish refrigerator (Figure 3). The tank was filled with fresh outside water from the Luga River estuary and refilled quarterly throughout the year (i.e., on 21 March, 21 June, 21 September and 21 December).

After a 3-month stay in the tank, 7–10 L of ballast water and sediments from a surface area of about 150 cm^2^ was pumped out with a sampler and filtered through a plankton mesh. Then, all the water from the tank was again renewed from the river with natural zooplankton and again remained there for three months.

Alive samples from the net were placed in anoxic conditions at +4 °C to terminate the diapausing stages and kill the active stages. Thereafter, diapausing stages were reactivated in accordance with above-mentioned protocol.

The sampler presents a rubber hemisphere connected to the end of a plastic tube equipped with low-power electric pump. A heavy iron ring is placed on the outside of the rubber hemisphere and serves to press the hemisphere to the lower deck in the ship’s ballast compartment. The rubber hemisphere has several small holes in the upper part for water inflow when the electric pump is on. The resting stages and other small particles are collected by water currents from the surface inside the hemisphere and are concentrated in a container through an outgoing tube and then filtered with a 50 µm mesh. A previous experiment in an aquarium with sand particles showed an almost clean bottom inside a hemisphere after filtering 10 L of water. The observation of organisms immediately after filtration did not reveal individuals damaged as a result of the action of the pump.

### 2.3. Design of Small-Scale Experiment

To confirm the time of formation of dormant stages, 3 buckets of 20 L each were filled with river water (containing zooplankton organisms) from the mouth of the Luga River 2 times a year: 21 September and 21 March, in parallel with the compartments of the ship. The buckets were covered with lids, placed inside the ship under dark conditions and stored at the temperature of the ballast compartment.

After 6 months of exposure, their contents were filtered through a plankton mesh and immediately observed under a microscope. Resting stages of crustaceans (calanid eggs, cyclopid larger copepodites and cladoceran ephippia) were counted and then reactivated according to the above written protocol. Organisms that survived after one week of anoxic exposure were considered diapausing stages that survived in conditions similar to the ballast compartments of a ship.

### 2.4. Statistics

All data were first checked with the Kolmogorov–Smirnov test for normality and then parametric (mean) or non-parametric (median) methods were applied to calculate average data.

## 3. Results

### 3.1. Invasive Species in the Ecosystems of the Gulf of Finland

In the summer, zooplankton and zoobenthos from the open and littoral parts of the mouth of the Luga River adjacent to the port of Ust-Luga, 72 taxa of aquatic invertebrates, or about half of the 145 species known from here, were identified [15] (Table 3). In general, the ratio of marine, brackish and freshwater taxa was expressed as 1:12:20, indicating a predominantly freshwater fauna in this region of the Gulf of Finland, where salinity rarely exceeds five ppm. In total, 11 species of invasive invertebrates were found. The largest proportion of invasive species in zooplankton was in the Copepoda class (14%) and in the benthos in Malacostraca (80%).

### 3.2. Seasonal Accumulation of Resting Stages in Ship Ballast Compartments

Resting stages of a few species were found in sediments of experimental ship ballast compartment: ephippia of *Daphnia cucullata* Sars, 1862, IV stage copepodites of *Mesocyclops leuckarti* (Claus, 1857), resting eggs of *Eurytemora affinis* (Poppe, 1880) and *E. carolleeae* Alekseev & Souissi, 2011, IV larval stages of *Chironomus plumosus* (Linnaeus, 1758). All these species were presented in our samples of zooplankton from June to August. Maximal density in sediments was found for experimental period lasting from September to December (Table 4). Dominant species were *D. cucullata* and *M. leuckarti* and the aquatic insect *Chironomus plumosus.* Mainly ephippia of *D. cucullata* and a few *M. leuckarti* copepodites IV were found in sediments in an experiment started in June. Dormant eggs of *Eurytemora* were presented in sediments created in December. No dormant stages were found in sediments of compartments filled with water in March.

**Figure 4:**
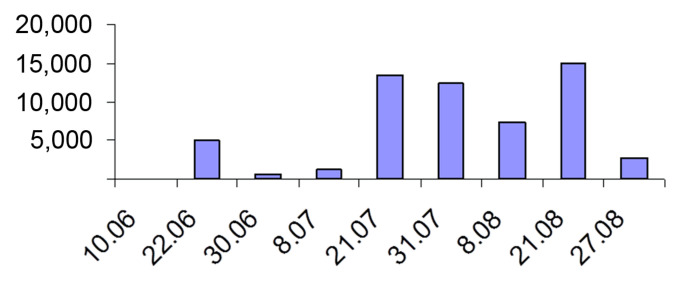
*Daphnia cucullata* Sars, 1862 population dynamics at Ust-Luga estuary, 2006 (density, ind/m^2^).

Small-scale experiments in 20 L metal buckets showed similar results: starting in September, they accumulated dormant stages of *Daphnia* sp., *Mesocyclops* sp. and *Chironomus plumosus* that could overcome 6-month stay in diapause; no dormant stages were created by zooplankton organisms collected in March (Table 5). Zooplankton in the estuary had typical population dynamics for this latitude, as indicated, for example, by *Daphnia cucullata* (Figure 4).

### 3.3. Dormant Species Biodiversity and Density in Sediments in Seaport Area after Reactivation

Copepods, cladocera, rotifers and nematodes dominated among the organisms recovering after dormancy. Additionally, the samples contained reactivated insect larvae (chironomids). Most sites were dominated by a few common species. At one site (Kotlin Island, Kronshtadt seaport area), recovering after diapause organisms, we discovered a new species for this climatic zone, *Metacyclops minutus* (Claus, 1863) (Copepoda). The total number of taxa identified from reactivated dormant stages was 18 taxa, where Rotifera (6) and Copepoda (5) dominated, followed by Branchiopoda (4). Nematoda (1), Ostracoda (1) and Insecta (1) were not identified to species level being larvae.

Density of reactivated dormant stages varied at large scales in sediments of all categories of waterbodies, except II (marine, fluctuating) that had no alive resting stages. The average density of resting stages for water bodies of different categories varied widely and was higher in the marine environment (category I). Maximal densities were noted in site #2, Kronshtadt (125,500 ind./m^2^), site #4, Neva River estuary (75,000 ind./m^2^) and site #3, Luga River estuary (65,000 ind./m^2^) (Table 2).

## 4. Discussion

### 4.1. Invasive Species in Ust-Luga Seaport Vicinity

In the vicinity of the Ust-Luga seaport, 11 invasive species were encountered, which had different origins (Table 3). According to the place of origination, the invasive species encountered by us are divided into North American (3), Ponto-Caspian (5), Southeast Asian (2) and Baikal (1).

*Metacyclops minutus*, known from Ponto-Caspian and Asian arid zones, was found for the first time in the Baltic Sea basin during the reactivation of dormant stages from sediments collected on Kotlin Island, the Gulf of Finland. Two more Ponto-Caspian species, *Pontogammarus robustoides* and *Chelicorophium curvispinum,* were also, for the first time, noted for the Gulf of Finland [16] (Malyavin et al., 2008). Another Ponto-Caspian invader, the mollusk *Dreissena polymorpha*, has been repeatedly recorded in the Gulf of Finland, which is considered one of the centers of its further dispersal to other regions of the world.

The predatory planktonic crustacean *Cercopages pengoi* is also associated with the Caspian Sea in origin but has long been noted as a common species in the Baltic fauna [3]. The bank of resting eggs of this species in the Gulf of Finland of the Baltic Sea, according to recent estimates, is up to 26 ∗ 10^3^ m^−2^ [17]. Introduced to America, it has long established stable populations in the Great Lakes of North America, where resting eggs are found in sediment layers dating back several decades [18].

Chinese Mitten Crab (*Eriocheir sinensis*), the origin of which is the Yellow Sea of China, has been repeatedly noted in the Baltic Sea, but in the Gulf of Finland, it was encountered for the first time [3]. *Acartia tonsa* from Southeast Asia estuaries was firstly recorded in the Baltic Sea in the first third of the last century [19]. This species should be listed as the first invasive copepod for the Baltic Sea.

*Gmelinoides fasciatus*, Baikal amphipod, was intentionally introduced in lakes of Karelia to improve fish food in the middle of the 1970s [20]. Via rivers connected with Ladoga lake, this species penetrated in this large lake where it almost fully substituted the native *Gammarus lacustris* [21,22]. Via the Niva River, it invaded the Gulf of Finland. In the time of our studies, *G. fasciatus* began invasion to the Luga River and moved 3–4 km each year [23]. Presumably, the superior ability of *G. fasciatus* to avoid fish predators contributes to its successful dispersal and the displacement of native species [24,25].

*Acanthocyclops americanus* and *Gammarus tigrinus* originally inhabited the North American coast and possibly arrived in the Baltic with ship ballast water [23,26]. Among the new species for the Baltic, one turned out to be new for science and was described under the name *Eurytemora carolleeae* Alekseev & Souissi, 2011 as an invader from North America.

At least a third of the invasive species we found in the Baltic are capable of forming resting stages that facilitate long-distance transport with ballast water. These include all copepods, as well as planktonic predatory crustacean *Cercopages pengoi*. All these species, in addition to being in plankton, were also reactivated in our experiments from sediments collected at the bottom of various water bodies in this region. This emphasizes the need to study the regularities of the formation of a bank of resting eggs in the areas adjacent to the seaport, which was carried out in the second part of our study.

### 4.2. Study on the Bank of Resting Stages of Aquatic Invertebrates

In the second part of our study, an experimental study of the seasonality of the formation of dormant stages in the area of the seaport was carried out, as well as a quantitative assessment of the accumulation of dormant stages in various parts of this part of the Gulf of Finland, including four types of main biotopes: temporary reservoirs associated with the bay; river biotopes of the Luga River; surf zone of the Gulf of Finland; and marine biotopes in the places where the forwarder of merchant ships of the Ust-Luga seaport and other ports of St. Petersburg pass.

To understand the process of resting-stage creation in nature, the timing of dormancy phenomena is important. In temperate latitudes, to which the study area belongs, there are two types of diapause, summer and winter [27,28], and there are some intermediate and overlapping cases [29].

In Harpacticoida, *Canthocamptus arcticus* is reported to diapause as resting eggs for 10 months from July to April [30]. *Heteropsyllus nunni* stays encysted during summer for 3–4 months and after excystment begins egg production within one week [31].

In the Cyclopoida, at least 34 species are known to have the ability to form resting stages, encysted or nonencysted, mostly as copepodid stages IV and V [32]. *Cyclops insignis* stays in dormancy as copepodid stages IV from April to December, while reproducing almost exclusively under ice [33,34]. Summer encystment is described in *Diacyclops* [35,36]. *Mesocyclops* and *Thermocyclops* show only winter dormancy as nonencysted copepodids, with the exception of the North American *Mesocyclops edax*, which develops a copepodid IV summer resting stage [37].

Marine calanoid resting eggs are usually released after the population declines from the plankton; this is in autumn in most of the species (*cf*. [38]).

In aquatic insects, for example, the genus *Chironomus* also has two types of diapause: summer and winter [39].

Our experiment in ship ballast compartment revealed the existence of two periods of resting egg accumulation: in June, this corresponds to the summer dormant stage (mainly in *Daphnia*) production and in autumn (September and December), when winter dormant stages in the all three groups of Crustaceans (Cladocera, Calanoida, Cyclopoida) and also in aquatic insects were found. This period of time should be evaluated as the most dangerous for alien species transportation with ship ballast water. Ballast water collected in winter and in early spring seems less dangerous for biological invasion.

It is possible to assume, and this variant was tested, that the conditions of the ballast compartment (darkness, deterioration in trophy, low temperature) themselves can contribute to the formation of resting stages, but as our experiments showed, this is not so. The seasonality of natural signals received at the end of summer and autumn, among which, at least for copepods and cladocera, the photoperiod plays a decisive role, are of paramount importance in the induction of diapause and, hence, the formation of resting stages.

It should be noted that a low-power pump was used during the collection of samples, and examination of the samples did not reveal damaged organisms. Thus, this sampling method can be assessed as adequate for the purpose of the study.

### 4.3. Spatial Distribution of Resting Stages in the Water Area of the Seaport and Related Parts of the Gulf of Finland

The spatial distribution of the number of resting stages was carried out at 10 stations, of which 5 were associated with the Ust-Luga seaport, and 5 stations in the overlying part of the Gulf of Finland, of which 3 were in deep areas associated with the movement of sea vessels and river-sea vessels. The only type of biotopes in which resting stages were not noted at all are shallow-water stations in the surf zone of the bay. A possible explanation for this can be considered the increased vulnerability of diapausing organisms associated with breaking waves, as well as winter freezing of this biotope. This negative effect is most likely associated with mechanical damage to the dormant stages.

In the port area, the highest concentration of resting stages was found at great depths in the area of the port of Kronstadt (125,500 ind./m^2^), in the estuary of the Neva River along the fairway of ships from the Gulf of Finland, moving in the direction of the White Sea–Baltic Canal (75,000 ind./m^2^) and in the area of the Ust-Luga Seaport on the traverse of the Luga River estuary (65,000 ind./m^2^).

Temporary reservoirs were characterized by noticeably lower maximum densities of resting stages of aquatic invertebrates (from 4680 to 37,440 ind./m^2^). At the same time, the greatest value is associated with the resting stages of rotifers and nematodes (the area of the Neva River). A temporary reservoir in the area of the port of Kronstadt, with low concentrations of resting stages, showed the highest level of diversity of organisms recorded. Finally, the river biotope of the Luga River, also associated with the seaport and the water that was used in our experiments on the seasonal variation of resting stages, demonstrated a relatively low level of concentration of resting stages (11,200 ind./m^2^). Thus, the greatest density of the resting stages in bottom sediments falls at greater depths, which is possibly explained by a larger water column, from which resting stages can settle, by runoff, part of the resting organisms due to autumn horizontal (planar) currents, as well as by the formation of perennial banks of resting stages due to the burial of part of the resting eggs of copepods and cladocera due to the processes of sedimentation of mineral and organic suspensions. It should be noted that this part of the bank of dormant stages remains viable for a long time (according to some sources, hundreds of years) [40]. When carrying out dredging operations, which are regularly carried out in the area of seaports and the fairway, this part of the buried dormant stages enters the water column and can easily be captured when filling the ballast compartments.

## 5. Conclusions

An analysis of the distribution vectors of invasive aquatic invertebrates found in the area of the Ust-Luga seaport makes it possible to identify three main sources of bioinvasions: North America, the Caspian Sea and Southeast Asia.

One-third of the invasive species found in this study are capable of forming resting stages, which facilitates their distribution.

The absence of living resting stages in the tidal zone of the Gulf of Finland apparently makes it difficult for waterfowl to capture and transfer resting stages during their spring migration.

Real-scale experiments in ship ballast compartments and smaller-size experiments in metal buckets revealed that the summer and late-autumn seasons have a higher potential of resting-stage accumulation in ship ballast compartment sediments and following transportation and dispersal of invasive species within at least six months (although, for other groups of crustaceans, this period can reach several hundred years).

One of the key findings of this study regarding the role of invasive species in the changing biodiversity in the Gulf of Finland is that they amount, today, to ca. 15% (11:72) of all identified taxa. This approximately corresponds to the average for the Baltic Sea for all groups of aquatic organisms, including fish and microalgae [3].

Accumulation of resting stages in various ecosystems directly connected with the water areas of the seaport indicates the potential danger for the spread of invasive species by ship ballast water, especially in the case of ballast water intake in this region. We consider *Acanthocyclops americanus*, *Cercopages pengoi*, *Dreissena polymorpha* and *Gmelinoides fasciatus* to be the most dangerous species among the found invasive forms. In addition to their negative impact on local ecosystems through predation and displacement of local forms, all four species are distinguished by a special ability for independent further dispersal after overcoming zoogeographic barriers with the help of human-mediated transfer.

Dredging leads to the mobilization of resting stages accumulated at great depths, which increases the risk of their capture by ships when filling ballast compartments. This must be taken into account to assess the risk of bioinvasion and to control the filling of ballast compartments in the area of such work.

## Figures and Tables

**Table 1:** Categories of sampling sites in Baltic Sea area.

Categories	Environment	Level of Stability	Type of Waterbody
I	Sea	Stable	Sea shelf, depth more than 4 m
II	Sea shore	Fluctuated	Sea tidal zone, dried lagoon, wind affected shelf
III	Continental	Stable	River
IV	Continental	Unstable/Temporary	Temporary waterbodies

**Table 3:** List of species of aquatic invertebrates found in the Luga River, Luga Estuary and reactivated from Gulf of Finland sediments. Invasive species indicated with bold font.

Taxa	Luga River	Luga Estuary	Sediments in Gulf of Finland
	Freshwater	Brakishwater/Sea	Reactivated
BRANCHIOPODA			
*Daphnia cucullata* Sars, 1862	+		+
*Ceriodaphnia quadrangula* (Müller, 1785)	+		
*Chydorus sphaericus* (Müller, 1776)	+		
***Cercopagis pengoi* (Ostroumov, 1891) (Ponto-Caspian)**		+	+
*Coronatella rectangula* (Sars, 1862)	+		+
*Bosmina coregoni* Baird, 1857		+	
*Podon intermedius* Lilljeborg, 1853		+	+
*Pleopis polyphemoides* (Leuckart, 1859)		+	
*Evadne nordmanni* Lovén, 1836		+	
*Leptodora kindtii* (Focke, 1844)	+		
COPEPODA			
*Pseudocalanus elongatus* (Brady, 1865)		+	
*Centropages hamatus* (Lilljeborg, 1853)		+	
*Limnocalanus grimaldii* (Guerne, 1886)		+	
*Temora longicornis* (Müller, 1785)		+	
*Eurytemora velox* (Lilljeborg, 1853)	+		
*Eurytemora affinis* (Poppe, 1880)	+	+	+
***Eurytemora carolleeae* Alekseev & Souissi, 2011 (North America)**		+	+
*Acartia bifilosa* (Giesbrecht, 1881)		+	+
*Acartia longiremis* (Lilljeborg, 1853)		+	
***Acartia tonsa* Dana, 1849 (Indo-Pacific?)**		+	
*Heterocope appendiculata* Sars, 1863	+	+	
*Eudiaptomus graciloides* (Lilljeborg, 1888)	+		
*Eudiaptomus gracilis* (Sars, 1863)	+		
*Ectocyclops phaleratus* (Koch, 1838)	+	+	
*Macrocyclops albidus* (Jurine, 1820)	+	+	
*Macrocyclops fuscus* (Jurine, 1820)	+	+	
*Eucyclops denticulatus* (Graeter, 1903)	+		
*Eucyclops macrurus* (Sars G.O., 1863)	+		
*Eucyclops serrulatus* (Fischer, 1851)	+		
*Eucyclops speratus* (Lilljeborg, 1901)	+		
*Cyclops strenuus* Fischer, 1851	+		
***Acanthocyclops americanus* (Marsh, 1893) (North America)**		+	
*Acanthocyclops vernalis* (Fischer, 1853)	+	+	
*Megacyclops viridis* (Jurine, 1820)	+	+	
*Microcyclops varicans (Sars, 1863)*	+	+	
*Mesocyclops leuckarti* (Claus, 1857)	+		+
*Thermocyclops oithonoides* (Sars, 1863)	+		
***Metacyclops minutus* (Claus, 1863) (Ponto-Caspian region)**			+
EUROTATORIA			
*Synchaeta baltica* Ehrenberg, 1834		+	
*Synchaeta fennica* Rousselet, 1909		+	
*Synchaeta monopus* Plate, 1889		+	
*Synchaeta* sp.	+		+
*Keratella cochlearis* (Gosse, 1851)	+	+	+
*Keratella quadrata* (Müller, 1786)	+		
*Keratella quadrata platei* Jägerskiöld, 1894		+	+
*Keratella eichwaldi* (Levander, 1894)		+	
*Euchlanis dilatata* Ehrenberg, 1832	+		+
*Euchlanis* sp.	+		
*Notholca striata* (Müller, 1786)	+		
*Lecane* sp.	+		+
*Polyarthra dolichoptera* Idelson, 1925	+		+
*Polyarthra minor* Voigt, 1904	+	+	
*Polyarthra remata* Skorikov, 1896	+		
*Polyarthra major* Burckhardt, 1900	+	+	
MALACOSTRACA			
*Gammarus lacustris* Sars, 1863	+		
***Gammarus tigrinus* Sexton, 1939 (North America)**		+	
***Gmelinoides fasciatus* (Stebbing, 1899) (Baikal)**	+	+	
***Chelicorophium curvispinum* (Sars, 1895) (Ponto-Caspian)**		+	
***Pontogammarus robustoides* (Sars, 1894) (Ponto-Caspian)**		+	
GASTROPODA			
*Viviparus viviparus* (Linnaeus, 1758)	+	+	
*Lymnaea stagnalis* (Linnaeus, 1758)	+	+	
BIVALVIA			
***Dreissena polymorpha* (Pallas, 1771) (Ponto-Caspian)**	+	+	
*Pisidium* sp.	+		
*Macoma* sp.		+	
MALACOSTRACA			
*Astacus astacus* (Linnaeus, 1758)	+		
*Pontastacus leptodactylus* (Eschscholtz, 1823)	+		
***Eriocheir sinensis* H. Milne Edwards, 1853 (Eastern Asia)**		+	
*Mysis* sp. juv.		+	
OTHER GROUPS			
*Hydra* sp.	+		
Ostracoda juv.	+		+
*Balanus* juv.		+	
Nematoda juv.	+		+
*Chironomus* sp. juv.	+		+
Odonata lar.	+		

**Table 4:** Seasonal variation in invertebrate resting stage accumulation in sediments of experimental ballast water tank, ship “Ostrov”, Ust-Luga port, St. Petersburg vicinity (mean ± standard deviation).

Date of Filling of Tanks	Dates of Draining of Tanks	Dates of End of Anoxic Termination	Copepoda Ind/100 L	Cladocera Ind/100 L	Insecta (Chironomus Larvae) Ind/100 L
21 September 2005	20 December 2005	February 2006	13.2 ± 1.2	6.6 ± 5.7	3.3 ± 1.7
21 December 2005	20 March 2006	May 2006	3.3 ± 2.1	0	0
21 March 2006	20 June 2006	July 2006	0	0	0
21 June 2006	20 September 2006	October 2006	3.3 ± 2.3	3.3 ± 2.3	0

**Table 5:** Seasonal accumulation of invertebrate resting stages in experimental 20 L metal buckets exposed to the experimental conditions on the ship “Ostrov” (mean ± standard deviation).

Date of Filling	Dates of Draining	Copepoda Diapausing, Ind/20 L	Cladocera Ephippia Ind/20 L	Insecta (Chironomus Larvae) Reactivated Ind/20 L
21 September 2005	20 March 2006	4.7 ± 1.2	6.7 ± 2.7	1.3 ± 0.3
21 March 2006	20 September 2006	0	0	0

## Data Availability

The data that support the findings of this study are available from the authors upon reasonable request.
